# The experiential health information processing model: supporting collaborative web-based patient education

**DOI:** 10.1186/1472-6947-8-58

**Published:** 2008-12-16

**Authors:** Laura A O'Grady, Holly Witteman, C Nadine Wathen

**Affiliations:** 1 Department of Health Policy, Management and Evaluation, Faculty of Medicine, University of Toronto, Healthcare, Technology and Place, Centre for Global eHealth Innovation, University Health Network, R. Fraser Elliott Building, 4th floor, Toronto General Hospital, 190 Elizabeth Street, Toronto, ON M5G 2C4, Canada; 2 Interactive Media Lab, Human Factors, Mechanical and Industrial Engineering, Health Care, Technology and Place, University of Toronto, 5 King's College Circle, Toronto, ON M5S 3G8, Canada; 3 Faculty of Information & Media Studies, The University of Western Ontario, North Campus Building, Room 254, London, ON N6A 5B7, Canada

## Abstract

**Background:**

First generation Internet technologies such as mailing lists or newsgroups afforded unprecedented levels of information exchange within a variety of interest groups, including those who seek health information. With emergence of the World Wide Web many communication applications were ported to web browsers. One of the driving factors in this phenomenon has been the exchange of experiential or anecdotal knowledge that patients share online, and there is emerging evidence that participation in these forums may be having an impact on people's health decision making. Theoretical frameworks supporting this form of information seeking and learning have yet to be proposed.

**Results:**

In this article, we propose an adaptation of Kolb's experiential learning theory to begin to formulate an experiential health information processing model that may contribute to our understanding of online health information seeking behaviour in this context.

**Conclusion:**

An experiential health information processing model is proposed that can be used as a research framework. Future research directions include investigating the utility of this model in the online health information seeking context, studying the impact of collaborating in these online environments on patient decision making and on health outcomes are provided.

## Background

A core element of the Internet has always been its capacity to facilitate communication. Designed to support the 'many to many' exchange mode, mailing lists served a similar function as that of newsgroups by providing a forum for asynchronous discussion by people with similar interests. Like email, these forms of computer-mediated communication (CMC) required use of software applications such as newsreaders or email programs. These applications became a popular medium to exchange information in the early days of the Internet. With the advent of the World Wide Web content became, in many cases, authored by 'one' source for 'many' to read ('one to many' mode). Once the web began to dominate as a preferred means to access content online various CMC tools were ported to web browsers to accommodate this shift.

Many organizations such as non-profits and hospitals, and in some cases individuals, utilized these technologies to support or enhance patient education. Some early online communities such as the Breast-Cancer List, now in its thirteenth year, are still in operation. Usenet health-related newsgroups are still available through the web interface Google Groups [1] with many active in both the alt.health and alt.support hierarchies. Consumer-driven web-based health bulletin boards and lists also exist; for example, WebMD forums [2], HealthBoards [3] and health-specific Yahoo Groups [4], although many of these may be considered relatively recent endeavours in comparison to their older cousins.

There are numerous and varied factors motivating use of the Internet as a means to access health care information. For some, the web has been used as a mechanism to both find and share information [5], to determine alternative or complementary treatments and for insights about rare conditions and new treatments [6]. For others, physical remoteness has led to Internet use to mitigate geographic boundaries [7]. For those seeking privacy, the ability to participate anonymously is a strong motivator [8]. In some cases clarification or further understanding of medical content is the goal [9]. One of the common elements found in these types of applications is the sharing of a personal story. Many health-related online forums have been used to provide anecdotal or experiential knowledge by individuals regarding various treatments or medications [5,10,11].

Early efforts to communicate online often fell victim to disruptive behaviour such as flaming, baiting or spamming. In other cases communities failed due to issues with the usability [12] or accessibility [13] of the interface, or with the sociability of the community [9]. For some health communities, a fading need may have been a mitigating factor. For example, the Crix List, a forum to exchange information related to the HIV protease inhibitor Crixivan, is no longer active. One of the main purposes of this list was to discuss the side effects of this new medication, and its demise may well have been due to growing understanding of interventions to reduce the impact of HIV/AIDS treatment side effects. These kinds of issues – alone or in combination – have likely contributed towards many online communities, including those on health-related topics, ceasing operation.

Recent advances and cultural shifts in online participation have coincided with technologies such as wikis and blogs that support this type of community sharing [14]. This shift, referred to as Web 2.0, includes the use of new tools that are designed to support collective knowledge sharing with interfaces that promote ease of editing and real-time changes unlike their web site predecessors [15]. Fewer technical barriers mean that those using these applications in order to learn about health are changing their roles from passively receiving information from a site (where content was often generated solely by the owner of the site), to collaboratively building knowledge [16]. This concept, where knowledge is generated amongst a group of individuals, has been described as the "wisdom of crowds," a notion that postulates the more people participating together to create knowledge, the better the information generated will be [17]. However, this idea raises concerns that learners with dissenting ideas and views may find such learning environments unfriendly. A collaborative filtering model in which popularity breeds popularity can lead to subject "icebergs," where less popular topics and ideas are submerged [18]. However, despite its potential pitfalls, the application of this concept in the domain of health remains a promising approach to the challenges of accessing useful information and support in a timely manner, and, many would argue, is an improvement on the pre-Internet days, when the capacity for group peer support was limited to the numbers of patients who could get together in person at any given time. Web 2.0 provides an environment that easily supports this type of behaviour, transcending to a significant degree space and time constraints. However, little research has examined the notion of collaborative behaviour in relation to health information seeking and knowledge creation on the Internet.

Collaborative web-based patient education is intended to encompass the use of web technologies that support information seeking in this context. This includes forums to exchange information, blogs that chronicle people's illness journeys, and wikis where a group of participants builds a repository of knowledge on a condition. Social networking web sites such as FaceBook and MySpace are also playing a role in these interactions. As the Internet has matured and made strides towards its original democratic ideals, collective knowledge-building using these applications has evolved. Implications for collaborative web-based patient education arise partly because this "social software" [19] helps participants to communicate and share knowledge directly with each other in increasing and novel ways. Online health information users can more easily bypass traditional information intermediaries such as medical professionals and instead use other community members as "apomediaries" [20] or health info(r)mediators [21], to learn about health topics, share and obtain recommendations about health providers and services at web sites such as Rate MDs [22] and Health Care Reviews [23], or even to collaborate in grassroots research by contributing and organizing personal health data at a site like PatientsLikeMe [24] or sharing personal genetic information at a site such as 23andme [25].

One example of knowledge building through collaboration is the DCA forum, a group of cancer patients taking the experimental drug DCA (dichloroacetate) off-label (i.e., not for its originally intended purpose) who share experiential or anecdotal information about using this drug [26], including exchanging dosage information and self-treatment protocols. Those new to off-label use of DCA are mentored by others more experienced in this application. Sharing this type of information by using the online technology allows others with cancer to learn about their illnesses [27].

However, as with all knowledge/information generated outside of specific standards or without the benefit of at least some expert review or guidance (even at the peer level), the issue of potential harms of misinformation must be considered. Processes of "misinfo(r)mediation" [28] highlight the fact that even well-intentioned provision of poor quality information can have significant negative consequences, and the Internet must continue to be seen as another "buyer beware" environment within the landscape of health information – one where the significant gains in access to, and transmission speed of, information can cut both ways – i.e., for high and low quality information. Concerns about misunderstanding and misuse of information are especially important in light of the expanding body of research in the areas of health literacy [29] and numeracy [30]. Skills for interpreting various forms of health information and integrating them into one's health decisions vary widely across populations [31]. Those with low health literacy and numeracy may be especially susceptible to misleading information and framing effects [32], whether these are intentional, as in malicious behaviour in an online community, or whether they simply reflect a poor fit between information content, its presentation, and the learner.

These issues provide a useful lens through which to examine health education and decision-making as complex issues for patients and physicians alike. For someone recently diagnosed with an illness, prognosis and treatment information are likely to be foreign and even daunting, requiring learning in the context of stress and perhaps fear. Making decisions in this context is a complex process that may involve a wide variety of interpersonal interactions, as well as information requirements.

Research to date indicates that there are a variety of roles in the patient/physician decision-making dyad. Physician-directed decision-making (stemming from the Parsonian model), while authoritarian in nature, was the primary means in health care delivery for many years. Various social movements, including feminism/women's health and other advocacy-based approaches, led to an emphasis on individual autonomy, and in the health realm, actively including the voice of the patient in a process of 'shared decision making' [33,34]. However, not all patients wish to be fully autonomous. Some may wish to be informed [35] and/or participate in certain kinds of decisions, but not others [36]. Charles et al. [33] proposed three primary models of decision making: the 'paternalistic' model where the physician makes the decisions, the 'informed or autonomous' model where the physician imparts knowledge to the patient and the patient makes the decision, and the 'shared decision making' model where the process is collaborative. The role assumed by a patient may have an impact on how information sources are weighted. For example, one study found that those who desired the most control in their decision-making stated that their physician was their main information source and many were guided by the doctor's preferences [37].

In addition to the patient-physician decision-making dyad there are also other possible collaborations that can affect this process. Physicians often consult each other to help with the decisions they make about care. In this provider-provider dyad, consultative practices start in medical school, continue during training, and are a method of information seeking and decision-making among practicing clinicians. Although not seemingly directly related to care, such interactions can be an important part of what treatment options are provided to patients, and in fact this practice has been cited as a barrier to evidence-based clinical care [38]. Information for all of those involved in care is an important element to the decision making process within this context.

People's information seeking behaviour (ISB) is complex and often iterative. Research in this area has produced consistent findings that comprise what has been called the "principles of information seeking" [39]; these include that people seek information 1) in familiar and comfortable patterns; 2) often following an informal to formal continuum; and 3) in an opportunistic and situated/contextualized way. Thus information seeking is often multi-faceted and complex and is comprised of interactions between individual, environmental and social factors. All of these variables and their interplay result in the observation that ISB is often seemingly "irrational" to the outside observer. Certainly these patterns are replicated in studies that have examined health information seeking in a variety of contexts [40,41].

Individual characteristics play a role in people's decisions regarding whether and how to seek information for a health condition. Some may be more likely than others to seek information as a coping strategy. For example, Miller's Behavioural Style Scale is predicated on the idea that people tend to cope with stressful situations by blunting and/or monitoring. Broadly speaking, blunters avoid information and prefer not to think about their stressful situations, while monitors actively seek information to reduce anxiety and help themselves cope [42,43]. Williams-Piehota et al. [44] demonstrated that for women at risk of breast cancer, adapting messages about the importance of mammography to receivers' behavioural style increased blunters' likelihood of obtaining a mammogram. In a study of metastatic cancer patients, Steptoe et al. [45] showed that while monitors had more factual knowledge about their health condition, they were less satisfied with the communication of their medical care. Although high levels of information seeking have been shown to be associated with effective coping strategies in cancer patients [46], other research has supported the idea that those who actively seek information may have poorer coping skills [43], a finding that should temper assumptions about the unvaryingly positive impacts of CMC in the context of health. In addition, individuals may themselves vary in their information seeking and coping styles, in some cases acting as blunters, while in others as monitors, and this may be due to contextual factors such as the person's understanding of the threat posed to them by the situation [47], and the type of stressor encountered [48].

Other information seeking theories examine motivation as a key aspect of information seeking and behaviour change. The Extended Parallel Process model has been proposed to explain how people rationalize decisions they make in relation to messages that evoke threat and fear, and how efficacy influences the ability to change [49]. For example, health promotion messages about the dangers of smoking (threat) are ubiquitous, and invoke concerns about cancer (fear), but individuals may continue to smoke because they do not think they are able to quit (lack of efficacy). Similarly, the Theory of Reasoned Action has been used to explain the ways in which individuals and groups engage in information seeking. For example, African American men have specific behavioural and normative beliefs in relation to seeking information related to prostate cancer that may differ from those of Caucasian American men [50]. Another theory, the Health Belief Model, attempts to explain motivation regarding behaviour in relation to goals and values. If someone places high value on their health, it is believed they will engage in behaviour to maintain it [51]. Other more comprehensive information seeking frameworks take into consideration other variables such as the source of information, mechanism, individual differences and external environmental variables such as cultural and socio-economic status [52]. These theories tend to explain motivation for seeking information but do not account for the desire to do so collaboratively or to find others in a similar circumstance in order to obtain anecdotal or experiential information.

Given these individual and situational influences on health information seeking, it is perhaps not surprising that people will use new media to explore their health conditions and their needs for both information and social support. The Internet can be seen as but one more way – and for some a particularly convenient and useful one – to meet these needs. Indeed it has been suggested that "sharing ideas and experiences with others through online health support groups may have health benefits." [53], and online communities have been described as the "...single most important aspect of the web with the biggest impact on health outcomes." [54].

We are now beginning to understand that these collaborations are an important element in supporting those learning about health conditions [55]. In particular, some patients may be seeking anecdotal information about their conditions from others who have the same condition but are not relying on online environments [56]. We believe that as Web applications such as wikis, blogs, and social networking sites continue to proliferate, more and more patients will be sharing and learning from each other in online environments. Increased participation in online communities strengthens the potential for patients to influence each other's decision making, emphasizing a third decision making dyad: patient-patient. It must be noted however, that, as described above, many of the information seeking patterns we now see on the Web are not in fact new – they merely replicate, in a new environment, the patterns and preferences for information seeking seen in non-online environments. What is new is the increased ability for some people to access "more people like me" in very fast and highly convenient ways. Therefore we must move towards a model that explains collaboration with other patients in health information seeking. We now shift our focus to consider ways in which these online environments can improve the capacity to support collaboration in relation to web-based patient education, in particular that which acknowledges experiential learning. We explore one theory of experiential learning as a way to understand the benefits, and potential harms, of online patient collaborations.

Experiential learning is defined as learning by doing or learning based on experience. It is often associated with informal adult learning such as life experiences occurring outside formal classroom instruction. Kolb [57] proposed a four stage cyclical process that includes concrete experience, observation and reflection, forming abstract concepts, and testing in new situations. Table 1 adapts this process to the patient education experience in a web-based environment. We refer to this framework as the experiential health information processing model.

**Table 1:** The experiential health information processing model

Kolb model for experiential learning	Steps in patient experience
1. Concrete experience: an event	The diagnosis of an illness, presentation of treatment options or other decisions related to care creates a need for information.
2. Observations and reflections: thinking about the event and its impact	By entering an online community an individual observes by reading the messages and reflects about their own experience in relation to the information shared.
3. Formation of abstract concepts and generalizations: what was learned	Inquiry through posted messages is made regarding a patient's next steps or treatment decisions related to their care from other community members.
4. Testing implications of concepts in new situations: active experimentation	By using knowledge acquired from the group the patient proceeds to a treatment decision.

By framing collaborative web-based patient education in the context of experiential learning we may find that those who participate in the forums are seeking specific forms of support from the community, particularly at stages 2 and 3 where requests for and presentations of new information play a key role in the learning experience. Participants who post their questions and concerns in an online forum may in part seek to have these issues addressed by other more experienced members [58]. Facilitators or seasoned participants may play a key role in ensuring that this occurs.

In many online forums participants focus on sharing their experiences, which in turn becomes filtered and distilled within the community such that the experience becomes de facto "information" or "fact". For example, someone may post a positive experience about taking a medication and be quite persuasive (intentionally or not) in suggesting that others will have the same successful outcome regardless of whether this is medically indicated or even possible. Indicators of trustworthy or believable content found online have included credentials of the author (e.g., a medical degree) [59], among others [60]. However, as more information is shared amongst laypeople online, the accredited status of 'MD' (or other traditional source credentials) may become less important. Individuals with a disease or condition are beginning to emerge as authoritative sources [61]. As more participants collaborate online, distinctions between a web site, its content, and other users becomes less defined. With this blurring, credibility indicators will likely shift as well. Those who are reading online content need to both determine the credibility of the message and its applicability to their own circumstances. Some may be unduly influenced or give credence to experiences shared anecdotally over more scientifically acceptable forms of information, due in part to the perceived credibility of the person posting the message. As the patient-patient dyad in an online context becomes more popular, credibility may become an important issue in relation to decision making in the offline world (e.g., between patient and physician).

A key step will be to test this model empirically. A number of research designs lend themselves to this task. For example, in depth qualitative interviews would be an important first step to understand whether the model, and it's proposed stages, can be applied to the context and experiences of these users – i.e., does the Kolb model map onto this learning context, and if not, where are the divergences? For example, did a diagnosis create an information need (Stage 1)? If so, did the patient explore an online community or other sources of information? If an online environment was used to meet these needs (Stages 2 and 3) then in what ways and to what extent? Similarly, focus groups with assorted types of participants in these communities (new, experienced, etc.) could elucidate group processes. Various participant-observation and/or ethnographic approaches, including analysis of postings, could provide a clear sense of these processes *in situ*. Finally, to understand the actual impact of these processes on important health (and other) outcomes, studies employing longitudinal methods would be an important second step in a proposed research agenda. For example, participants would be asked to what extent and how the information obtained from others online was useful or influential, and, conversely, whether decisions made using these processes were later regretted (Stage 4). The findings of these types of research could have important implications for those who design and support such environments, and also for our understanding of processes of learning in new media environments more generally.

Understanding at what stages an individual requires information could provide important insight into both individual outcomes, as well as sustaining the community. For example, if it is understood that most new learners require a period of time in which they prefer to only read messages online before actively participating, this could be outlined in the instructions for participation. Of equal importance will be examining disruptive behaviour within the model. Disruptive behaviours in traditional collaborative web-based environments such as forums and lists include spam (unsolicited messages), flaming (messages that attack others), baiting (an inside joke in which participants are solicited to post responses that are humorous to others) and trolling (messages that contain false responses to an inquiry, generally to provoke an argument) [9]. Blogs can also be subjected to these types of behaviours through the comment section, while in applications such as wikis, malicious altering of content is also a concern [16]. Research directions in this area include investigations about how disruptive behaviour in online communities might affect an individual's search for health information and what mitigation and management techniques for dealing with this are most effective and appropriate in collaborative web-based patient education communities.

Another avenue of future research is to explore potential clinical applications of experiential patient engagement and learning in online environments. Individual stories that are propagated using collaborative applications through popularity ratings may promote learning but may also effectively submerge information that has in fact been verified by formal research. By tracing the online spread of such ideas via network analysis, models of online information flow in collaborative Web applications could be developed.

Sharing experiential information through blogs and other mechanisms as compared to other methods of conveying information online may change the way information is acquired, perceived, and internalized. In addition, investigations that compare these approaches to other forms of information exchange, including in-person support groups, and more self-directed, non-social approaches to information-seeking and learning are required. This is a rich area for research that has yet to be well-explored.

Also of interest is the evolving nature of credibility and the way it is depicted, understood and accepted as more laypeople become recognized as experts and opinion leaders in online environments. More research will need to be conducted in order to understand this evolving concept. In this area, quantitative and visual methods such as social network analysis offer tools for analyzing the social nature of learning. Such analyses may be strengthened through the use of now-ubiquitous Web features in which users rate or comment on each other's content. Feedback to commonly seen questions such as, "Was this review helpful to you?" can provide proxy measures for assessing the impact of individuals within a collaborative learning environment.

The face of health care on the Internet is changing. Just as we begin to research how new technologies are being used by health-interested web users, they change and impact the frame of reference. Recently, large scale web-based personal health record repositories have been implemented by Google (Google Health) and Microsoft (Vault). It remains to be seen whether or not these initiatives will eventually supersede many of the smaller, community or hospital-based projects that currently support peer to peer exchange of information. However, it is important to understand user needs and behaviour when implementing any technology, including that which supports collaboration in web-based patient education.

Many theories exist that attempt to explain information seeking, and some have been applied to health-related scenarios. However, few address this behaviour in relation to others that seek information or those who are specifically looking for peer experiences, guidance and support. Since most (if not all) individuals searching for information about their health care condition are already motivated (as defined by actively seeking help) we propose, in contrast to models based on motivation, an online health information seeking model based on learning theory. A key to understanding and supporting collaborative web-based patient education will involve examining environments that aid such information exchange using tools, technologies and approaches that assist these processes. Knowledge about an individual's health condition that is constructed collaboratively through collective sharing of experiences can provide not only "information" but also support, and, a key for many health information seekers, care [62,63]. Given that these forms of collaboration may well influence the decision making process for patients, we need to consider ways to better enable and support the exchange of experiential and anecdotal information, and help patients differentiate the different kinds of information to which they may be exposed in these environments. This is particularly important in a rapidly evolving technical environment, where we need to find ways to test whether new technologies actually help people, and if so, in which populations and in what ways. We also need to be cautious and critical about the as yet untested benefits of these emerging technologies. We must be mindful that contribution rates in these online environments remain low, with lurkers (those who read messages but do not contribute) continuing to significantly outnumber more active participants.

If collaboration is indeed an important element to patients' online health education experiences a key next step will be finding ways to understand and, ultimately, support experiential learning, and reinforce the capacity for individuals to learn in these environments by enabling their exposure to others with similar needs and experiences. It is important to note that despite rhetoric about the democratization of information via the Internet, online interactions may simply reproduce existing power structures and may not, in fact, truly empower patients [64,65]. We propose that understanding each step of our proposed experiential health information processing model will help elucidate the interactions between users and other users, mediated by the technologies. Delineation of the steps outlined in Table 1 can assist organizations and individuals working in the field of patient education. Additional research – perhaps following approaches taken to other complex and multi-faceted socio-technical (so-called "wicked") problems [66] – is urgently required.

## Competing interests

The authors declare that they have no competing interests.

## Authors' contributions

LOG conceptualized and wrote the first draft of the paper. HW added content throughout the paper, contributing most substantially to the sections on determinants of health information seeking behaviour, the role of new technologies in information seeking behaviour, the future directions and conclusions. NW wrote sections related to collaboration in web-based patient education component as well as the future directions and conclusion sections and contributed substantially to the content of the remaining sections. All authors read and approved the final manuscript.

## Pre-publication history

The pre-publication history for this paper can be accessed here:


